# Prediction in forensic science: a critical examination of common understandings

**DOI:** 10.3389/fpsyg.2015.00737

**Published:** 2015-06-02

**Authors:** Alex Biedermann, Silvia Bozza, Franco Taroni

**Affiliations:** ^1^Faculty of Law, Criminal Justice and Public Administration, School of Criminal Justice, University of LausanneLausanne, Switzerland; ^2^Department of Economics, Università Ca'Foscari VeneziaVenice, Italy

**Keywords:** prediction, prevision, probability, statistics, forensic science, forensic psychology

## 1. What is “wrong” with prediction?

The term “prediction” is commonly used throughout science. As part of expert language, however, the term holds a partially conflicting status because its meaning is not always properly perceived among both scientists and recipients of expert information. In judicial proceedings, for example, the meaning of “prediction” may be that of more informal language and hence be misread as a definite assertion. This is a cause of concern because it misconceives an essential aspect the actual state of affairs, that is uncertainty.

In his comprehensive text *Theory of Probability* (1974), Bruno de Finetti explicitly highlighted a warning (de Finetti, 1974, [p. 98])^1^:

“Think: prevision is not prediction!”

By *prediction* de Finetti was referring to assertions of certainty about possible alternatives that, in reality, are unknown to us. Actually, the *possible* alternatives typically refer to past, present or future events that are *not known* to us with certainty. Take, for example, the event “It will rain tomorrow at noon in the city center of London.” As a statement today, this would amount to an ascertained truth, along with a “venture to try to ‘guess,’ among the possible alternatives, the one that will occur.” (de Finetti, 1974, [p. 70]). de Finetti noted that such statements are “often made, not only by would-be magicians and prophets, but also [at times] by experts” (de Finetti, 1974, [p. 70]) ^2^ in scientific and commercial fields. He further argued that making statements about things that are uncertain to us should, instead, be called *prevision*, a term which “consists in considering, after careful reflection, all the possible alternatives, in order to distribute among them, in the way which will appear most appropriate, one's own expectations, one's own sensations of probability” (de Finetti, 1974, [p. 72]). The distinction between prevision and prediction was a common important theme throughout his writings, both mathematical and philosophical (e.g., de Finetti, 1983).

The fact that the uncritical use of “prediction” arises in connection with experts in many important fields is worrying, and merits discussion. In forensic science, for example, the term prediction is typically encountered in connection with the study of the relationship between results of DNA analyses (e.g., of single-nucleotide polymorphisms) and externally visible characteristics (EVC), such as eye and hair color. This is a lively area of research in applied genetics, with increasing interest among forensic scientists. In particular, we may be confronted with legal cases in which a database search of a conventional DNA profiling result does not lead to a correspondence based on short tandem repeat (STR) markers. As a result, no potential donor for a crime stain can be provided (e.g., Kayser and Schneider, 2009; Walsh et al., 2014), but leads regarding EVC. Moreover, the concept of prediction is encountered almost endemically in the area of forensic anthropology. For example, one reads common allusions to “sex/age/stature prediction” based on measurements of various body parts; or in the analysis of illegal substances, the prediction of the outcome of additional analyses based on previously analyzed items. In forensic psychology, prediction is used in discussions and the assessment of the behavior of individuals (e.g., Douglas et al., 2003).

In this brief comment, we shall take a closer look at exemplary applications in forensic science, in particular applied genetics, where the term prediction is now used prominently and increasingly often. Section 2 will explain relevant subject matter on what in current statistical literature is known as a “predictive distribution.” This background will be used, in Section 3 to discuss the notion of prediction with respect to forensic applications, and to examine the extent to which the common use of this term in forensic science is or is not well founded. Section 4 will discuss implications of such foundational understanding of terminology and concepts for the practice of logical, balanced and transparent reporting in forensic science applications. The discussion will concentrate on forensic science and statistics (corresponding to the expertise of the authors), but the subject matter is similarly encountered in many other fields such as medical diagnosis and other matters in which the use of scientific results has direct societal impact. Without loss of generality, this also includes the theory and practice of psychology, in particular forensic psychology.

## 2. Predictive distributions in Bayesian statistical theory

Prediction is a clearly defined term in standard statistical literature, in particular among adherents of the Bayesian point of view which is favored in this article. Formally, we can distinguish two main elements. The first is past observations, denoted as data such as *x*_1_, …,*x_m_*. The second element is future observations such as *y*_1_, …,*y_n_*. Our interest is to represent beliefs about possible values of the future observations—in terms of probabilities, conditional on the given past data. We follow several writers in using the short-hand notation *x* and *y* for past and future data, respectively. This order naturally translates the temporal order of the data, although it is worth emphasizing that the future data *y* are deemed to be informative about *x*, but *not* in the sense of common allusions to “repetitions of an identical experiment” (Lindley, 2000).

The probability distribution of uncertainty regarding past or future observations is often asserted in one of standard known forms. This is typically denoted by *f*(· | θ), in which the only unknown feature is the parameter θ. The related probabilistic model is known as a parametric model, and is denoted by {*f*(· | θ), θ ∈ Θ}. In the Bayesian paradigm Θ represents a parameter set (e.g., Robert, 2007) which allows for the assertion of further available information in terms of a so-called prior distribution, usually denoted by π(θ). It summarizes the way past values of *X* are asserted to inform us about possible values of *Y*.

Statistical inference is performed by computing a posterior probability distribution for θ, usually denoted π(θ| *x*). Statistical inference about *Y* on the basis of observing *X* = *x* is achieved by the further computation of the mixture distribution

(1) $$\begin{array}{l} f(y|X=x)\;=\;\int_{\Theta }f(y|\theta )\pi (\theta |X=x)d\theta \text{ for the}\\ \text{ various possible values }y. \end{array}$$

**Figure 1:**
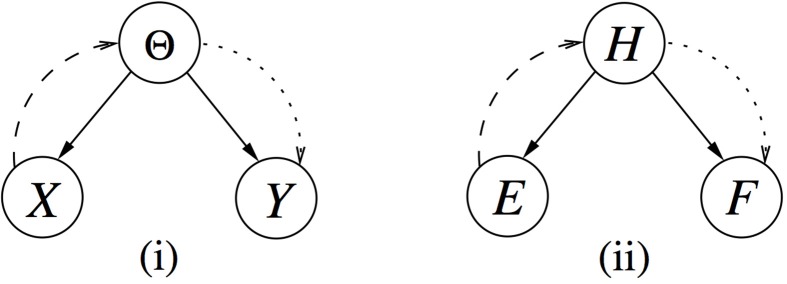
**Bayesian networks with variables ***X*** and ***E*** denoting past data, and ***Y*** and ***F*** denoting future data**. Both past and future data depend on and are related through, in **(i)**, parameter θ, belonging to the parameter space Θ, and in **(ii)**, a variable *H* denoting discrete hypotheses. The dashed edges denote the process when going from past data to the parameter, called *inference*, whereas the dotted edge illustrates the process of going to future observations.

To clarify this understanding, consider the graphical model [i.e., a Bayesian network (Cowell et al., 1999)] shown in Figure 1(i) where, by convention, variables are denoted by capital letters. In particular, let *X* denote the past data, with the corresponding lower case letter *X* denoting a *realization* which is the observed value of *X*, as introduced above. The inferential process can be described by the dashed curved edge. By summarizing our uncertainty about *Y* as informed by *X* via θ, we complete the assertion of *prevision*. Stated otherwise, our prevision is an assertion of uncertainty about possible but unknown values of *Y*, here summarized by an expression of our personal uncertainty in terms of π(θ | *x*).

Another goal in statistics consists of contemplating about a future observation *Y*, where our belief structure is again described according to the parametric model *f*(· | θ) above, that is *f*(*y* | θ). The parameter θ is unknown, but one can incorporate posterior knowledge about θ (i.e., including current observations *X*) and compute a probability distribution *g*(*y* | *x*). It has the form of a weighted average of all possible distributions on *Y*, *f*(*y* | θ), with the corresponding distribution describing personal uncertainty about θ, that is *g*(*y* | *x*) = ∈ *f*(*y* | θ)π(θ | *x*)*d*θ. This is shown in terms of the dotted edge in Figure 1(i), where capital letter *Y* denotes the future data, with the corresponding lower case letter *Y* denoting a realization. The passage from the parameter θ (or θ including *X*) to a future observation *Y*, is referred to as *prediction* in (e.g., Robert, 2007) or, following presentations in the close spirit of de Finetti's development, *prevision* (e.g., Lad, 1996). So, *g*(*y* | *x*) is typically called a *predictive distribution*, but again, what we mean is a *prevision*, that is a summary of our personal uncertainty about the outcome *Y*, using probability. There is no statistical support for the notion of prediction as an assertion of certainty about heretofore unknown observations.

It is also possible to consider a discrete variable (i.e., hypothesis) instead of θ, denoted *H* short for “hypothesis” [shown in Figure 1(ii)] or *T* short for “theory,” as encountered in general scientific reasoning (e.g., Press, 2003). The variables *H* or *T* can have two ore more possible realizations. They are in exactly one of their possible realizations, but which one may be unknown to us. Note also that in forensic statistics, it is common to use the notation *E* (short for “evidence”) (e.g., Aitken and Taroni, 2004). The predictive distribution for the discrete case can be derived in a similar manner, as it will be shown by means of a practical example in Section 3. With these theoretical elements in mind, we can now move on to examine the understanding of the term prediction in selected areas of application.

## 3. Forensic inference, not prediction

Consider the following scenario: biological (trace) material from a donor with unknown characteristics is available. DNA profiling analyses are conducted and results are used to help inform about features of the donor of the analyzed material. The features of interest may be hair and eye color, for example. Is this a situation requiring *prediction* as is commonly suggested (e.g., Pośpiech et al., 2012), more or less informally in many scientific communications on this topic? According to the statistical account outlined in Section 2, such analysis should properly be called *inference* or, more generally, *prevision*.

This conclusion can be understood through the following reconstruction. The results of DNA analyses on a trace item from a donor whose EVC is unknown represent the available forensic data *E*. These data form the basis for making an *inference* about *H*, that is various competing propositions regarding the EVC of the donor of the analyzed material. Thus, the focus is on *Pr*(*H* | *E*), that is the posterior probability of *H* given the scientific finding *E*, obtained through a standard application of Bayes' theorem. This probability distribution for the various possible states of the variable *H* represents our *prevision*. We do not make a prediction on what we think what EVC actually holds.

The notion of prediction arises with another aspect of the current scenario, in particular if we turn our attention to a future observation. Continuing the logical temporal order in notation with respect to *E*, let such a future observation be denoted *F*. It could consist of, for example, the analysis of another genetic marker, or set of markers, on material that comes from the same person as that for which results *E* are available. In graphical statistical terms, this can be translated as a diverging connection *E* ← *H* → *F*, where the probability distribution for *F*, conditional on previous data *E*, is called the predictive probability distribution [Figure 1(ii)]. It is given by $Pr(F|E)\;=\;\sum_{i}^{n}Pr(F|H_{i})Pr(H_{i}|E)$, when there are *n* distinct hypotheses regarding the donor's EVC.^3^ The expression is also known as the *(posterior) predictive distribution* because it includes knowledge of the previous experiment *E*. Without *E*, it would reduce to the prior predictive distribution (or, marginal distribution) of *F*, written $Pr(F)=\sum_{i}^{n}Pr(F|H_{i})Pr(H_{i})$. Use of such predictive distributions is typically made in forensic attribute sampling, when assessing the probability of additionally analyzed items drawn from a consignment of items (e.g., Biedermann et al., 2008). In the context of our discussion here, the important qualification regarding the term “prediction” is that the inference performed supports a “predictive *distribution*,” that is, a probabilistic distribution of our uncertainty over the possibilities given all available evidence.

Similarly, the formal framework of analysis (Figure 1) can capture reasoning patterns that are commonly encountered in forensic psychology. Suppose, for example, that past observations *E* regarding particular behavior (e.g., violence) need to be used to make statements about potential future behavior *F*. If one can accept that this psychological assessment task exhibits enough structure, then the generic framework exposed in Section 2 allows one to describe this task in precise terms, extract information from these descriptions, and transform this information into conclusions. More generally, the framework represents an instance of a normative approach by providing a reference point against which actual practice may be compared (e.g., Baron, 2012), and by guiding reasoning research (e.g., Crupi and Girotto, 2014).

## 4. Discussion and conclusions

In statistical theory, the term “prediction” typically arises in connection with so-called predictive distributions that summarize a reasoner's belief about future observations. de Finetti dismissed the term because of its potential suggestion of a definite conclusion when, in reality, the reasoner's state of knowledge is incomplete (i.e., one of uncertainty). He considered assertions affected by uncertainty, expressed through probability, to be more appropriately termed “previsions.” This term is more general as it applies to any announced probability distribution. That is, it is not restricted to future observations, but can apply to past occurrences about which we are uncertain too.

To some extent, this understanding is at odds with uses of the term “prediction” in applications such as forensic genetics where the target of evaluation is a conditioning hypothesis *H*, which is a current state of nature (e.g., regarding a person's EVC). Summarizing our uncertainty about the possible states of a conditioning hypothesis *H* is a case of prevision. It is not a prediction in the sense of a statement about the state of nature that actually holds. It should be observed, however, that a scientist's conclusion is often used within wider contexts, such as judicial proceedings, where the meaning of “prediction” may be that of more informal language and hence be understood as a definite assertion. Such a misreading of the term prediction should be a cause of concern because it misconceives the actual state of affairs, that is a situation characterized by uncertainty.

To avoid this kind of complication, and where the object under investigation is the (posterior) probability of a particular hypothesis *H* (e.g., regarding a person's EVC, disease status, etc.), given results of forensic examinations *E*, forensic scientists could consider the option of referring to the reasoning process more generally as *inference*, not prediction. Nonetheless, in forensic contexts, this should not be taken as a suggestion that forensic scientists ought to provide inferential conclusions regarding hypotheses of interest. There are both procedural (e.g., role of the scientist in the legal process) and conceptual (e.g., inappropriate position to justify a prior probability) reasons for this (e.g., Redmayne, 2001; Aitken et al., 2010). Instead, according to current understandings in the field, scientists can help make the most reasonable use of probability by providing recipients of expert information with a likelihood ratio (e.g., Aitken and Taroni, 2004). This kind of expression informs other participants in the legal process about how they ought to revise *their* prior beliefs regarding the competing hypotheses of interest, whatever they may be.

### Conflict of interest statement

The authors declare that the research was conducted in the absence of any commercial or financial relationships that could be construed as a potential conflict of interest.

## Footnotes

^1^Textbox and italics as in original.

^2^Text in brackets added by the authors.

^3^Note that, for the ease of argument, only the discrete case is considered here.
